# Equity of access to free maternal and child health services among reproductive-age women in Delta State, Nigeria

**DOI:** 10.4314/gmj.v58i3.6

**Published:** 2024-09

**Authors:** Christie A Enuku, Obinna Onwujekwe

**Affiliations:** 1 University of Benin, Department of Nursing Science Benin City, Edo State, Nigeria; 2 University of Nigeria, Faculty of Medical Sciences, Department of Pharmacology, Enugu, Nigeria

**Keywords:** equity, educational status, geographical area, maternal and child health, socioeconomic status, Nigeria

## Abstract

**Objective:**

The study aimed to assess the equity of access to free maternal and child health services among reproductive-age women in Delta State, Nigeria

**Design:**

the study adopted a descriptive cross-sectional survey design

**Participants:**

The population for the study were 368 women of reproductive age (15-49 years) who had given birth between April 2015 and December 2015 in two randomly selected senatorial districts of Delta State. Data were collected using a pre-tested interviewer-administered questionnaire.

**Results:**

368 women were recruited for the study, consisting of 73.3% (N =270) from the urban setting and 26.7% (N=98) from the rural setting. 54.1% (N=199) had completed secondary school, while 28.8% (N=106) had completed tertiary education. Most respondents were aged 21 to 30 years 217(59.0%). 20% of respondents belonged to the poorest, poor and least poor socioeconomic status (SES) groups and 19.8% to average poor and rich SES groups. The results showed equity between different SES (0.014) and educational (0.027) backgrounds, as indicated in concentration curves. However, the concentration index between the geographical areas (-0.0200) indicates inequity in access in favour of urban dwellers.

**Conclusion:**

Free maternal and child health services (FMCHS) were equitable across the different SES groups and educational levels. However, there was inequity in access due to distance to the hospitals among the women of childbearing age in the communities. This study is relevant to all healthcare professionals, especially those in public health, because it will encourage them to exercise their energy towards home care to reduce maternal and child mortality.

**Funding:**

None declared

## Introduction

Improved maternal and child health is imperative for the survival of every nation and global population. Maternal mortality is on the decline in Africa, but sub-Saharan Africa still has the largest burden, including Nigeria.^1^ According to the World Health Organisation, the maternal mortality ratio dropped by 38% globally between 2000 and 2017.^2^ Low-income countries, including those in the sub-Sahara region, continue to contribute to more than half of all maternal deaths (LMICS).^2^ The slow improvement in maternal and child health outcomes in sub-Sahara Africa can be attributed to discrepancies in health-seeking behaviour and low utilisation of health services.^3^ Also, countries in sub-Sahara Africa, of which Nigeria is a member, continue to display persistent inequity in access to and utilisation of health services with significant differences in access between poor and non-poor populations.^4^,^5^

Wealth-related inequity remains high, with socioeconomic status and place of residence (urban and rural) being the main drivers in differences in utilisation of the services.^5^ Equity in the use of healthcare is defined according to two major classifications: vertical and horizontal equity^5^. Vertical equity means providing unequal healthcare needs to individuals with unequal healthcare needs. In contrast, horizontal equity is the notion that persons with similar health conditions and needs should be given equal treatment irrespective of their income and socioeconomic status.^6^ Inequities in the distribution of healthcare services is gaining global attention in public health.^7^

Access to maternal and health services is more challenging for poor individuals living in rural areas than in urban areas. Access is interpreted as freedom to use healthcare services without barriers. This is addressed from three dimensions: availability (physical access distance), affordability (financial access) and acceptability (cultural access).^8^

A retrospective study on equity of access to maternal health in Interventions in Brazil and Columbia revealed persistent pro-rich inequities in access to four maternal health systems.^9^ Also noted is a study on the disparity in maternal, newborn and child health services in high-focus states in India: a district-level cross-sectional analysis. The study revealed a significant disparity in the usage of maternal and child healthcare services in the district of India. Resource-rich people (urban residents and richest quintile) are way ahead of marginalised people (rural residents and poorest quintile) in the usage of health care services.^10^

A study to assess equity and access to maternal and child health services in Ghana revealed that geographic location, income status and formal education are key drivers of maternal and child health inequities.^11^ It was recommended that the government partner with the private sector to implement health policies to address inequity in maternal and child health (MCH) services through primary healthcare and resource allocation skewed towards rural areas and the lower wealth quintile to bridge the inequity gaps and improve MCH outcomes 11. Similarly, it was observed that high level of socioeconomic inequity with pro-rich in antenatal and skilled delivery in private facilities in Namibia revealed that women with high wealth status and education have a 70% utilisation level of antenatal and skilled delivery compared to the poorest with 47% utilisation rate.^12^

Another study conducted on inequity in maternal and child health utilisation in Nigeria revealed that there was higher and increasing inequity in maternal healthcare. In contrast, inequity in utilisation of child healthcare decreased over time, with health status and education as the major non-need drivers of inequity in utilisation.^13^ Inequity in access to health care was identified as one of the causes of maternal and child mortality in the Delta state. To equitably decrease maternal and child mortality in Delta State, Nigeria, the Delta State Government established free Maternal and Child Services.^14^ Another study in China revealed an inequity of MCH care in the utilisation of essential public health services programmes 15 Furthermore, a study conducted in sub-Saharan Africa showed unequal access and utilisation of maternal, newborn and child health services. A special focus on urban settings revealed variations in maternal and child health services utilisation across urban sub-Sahara Africa(SSA), which are inequity in the urban settings of the region, including poverty, low level of education, unemployment, low socioeconomic status and young maternal age 16. Horizontal inequity is what the government of Delta State intended to reduce. Hence, the inequity is tailored to maternal and child health services because reproductive-age women tend to have similar conditions and needs and should be given equal treatment irrespective of their socioeconomic status and where they live, be it urban or rural. It was hoped that the FMCHS would ensure equitable utilisation of MCH services for all population groups in the state.

FMCHS is based on a strategic plan developed for equitable access. The free services provided covered antenatal care and delivery, including Caesarean section, Laboratory tests, Blood transfusion, post-abortive complications, Ectopic pregnancy, and Incidental ailments. However, since the inception of the FMCHS in Delta State, no evaluation has been conducted to assess the extent to which the services achieved their equity objective. It is unclear the level of equitable accessibility of the services to reproductive-age women in rural and urban areas, to all socioeconomic groups, and people of all educational statuses in Delta State. Thus, this study assessed equity of access among reproductive women who utilise free maternal and child health services in Delta State. The specific objectives were to assess equity in access to free maternal and child health services between reproductive women of different socioeconomic and educational statuses in Delta State, Nigeria.

## Methods

### Study Design

This study was a community-based, cross-sectional study design. The study populations were women of reproductive age (15 – 49 years) who had given birth between (April 2015 and December 2015) in the three senatorial districts of Delta State. At this stage, they attend antenatal or post-natal clinics at the hospitals in Delta State Free Maternal and Child Health care programmes.

### Study Area

The study was undertaken in Delta State, South-South Nigeria. Delta State was created on 27 August 1991 from the then Bendel State. It has an area of 17.698 Km^2^.^17^. Delta State is ethnically diverse, with people who speak numerous languages. The State is divided into two main groups based on historical relations, culture and language. The population of Delta Central is 1,575,738, Delta North Senatorial District is 1,229,074, and Delta South Senatorial District is 1,293,479, respectively.^15^

Delta state is made up of twenty–five local government areas with sixty-one secondary health facilities and one tertiary health facility.^14^

### Sampling technique and Sample size determination

The sample size determinants for the household survey were done using the Taylor formula, which was found to be 368, where P = the prevalence of the problem (the prevalence of antenatal care attendance from a previous study in Nigeria was 60.3%).^18^,^19^, respectively. A multistage sampling method was adopted for the study. Stage I: Two of the three senatorial districts were selected using a simple random sampling technique of balloting. This was done by generating a sampling frame, which is as follows: The researchers begin by identifying the names and boundaries of the three senatorial districts in the state and assigning identification numbers.

A unique identification number was assigned to each senatorial district. (S1, S2, and S3) to represent the three districts. Then, the researchers created a list that included each senatorial district and its corresponding identification number. This list served as the basis for the sampling frame. Confirm that all senatorial districts listed in the sampling frame are accessible for sampling purposes. The researchers ensured no legal, logistical, or administrative barriers to accessing districts. The sampling frame was used to select two senatorial districts randomly. With the finalised sampling frame, a random number generator or a similar randomisation method was used to select two senatorial districts randomly. These selected senatorial districts were Delta South and Delta Central senatorial districts. The two senatorial districts have eight LGAs each.

Stage II: Two local government areas were randomly selected from the two senatorial districts using a simple random sampling method (lottery method). The local government areas selected were Isoko North from Delta South and Ughelli North Local Government from Delta Central.

Stage III: From the two local government areas selected from Stage II, two communities with hospitals were from each local government area, making it four communities. These communities are Ozoro and Ofagbe from Isoko North and Ughelli and Ewu from Ughelli North.

Stage IV: from these communities selected above, respondents were selected for the study using a purposive sampling technique based on the following criteria: respondents must have given birth between (April 2015 and December 2015, and the baby must be less than one year old within the survey period. The index house where the first respondent was selected was spun in the estimated centre of the communities. The direction of the bottle after being spun determined the starting point of sampling.

Cochran formula^20^ was used to assign the sample to strata, which is in the approach of proportionate stratification. Using the Cochran formula for the household survey, 368 sample sizes were proportionately distributed to 128 households in Ozoro, 46 in Ofagbe, 142 in Ughelli and 52 in Ewu. The number of households was obtained from the immunisation unit of each local government council concerned. The household population for Ozoro is 45,271, Ofagbe 16,050, Ughelli 60,101 and Ewu 18,220.

### Study instrument

A pre-tested interviewer questionnaire adapted from Onwuyekwe et al. with close-ended questions.^21^ The Household Interviewer Questionnaire was divided into three sections A to C. Section A contained information on socio-demographic data about respondents and their households. Section B: to ascertain the utilisation of maternal and child health services among different socioeconomic groups, educational backgrounds and geographical locations among reproductive-age women. Section C: to assess equity in access between different socioeconomic and educational statuses, as well as rural and urban areas of the reproductive age women receiving free maternal and child health services. A mother was considered to have used a service if she had accessed it at a health facility at least once. In the case of delivery services, she was considered to have used the service if her last delivery was at a health facility. She was considered to have used immunisation services if requested at a health facility.

### Validity and reliability of Instrument

The statistician, maternal and child health experts, and health economists ascertained the instrument's face and content validity. A test-retest reliability pilot test was conducted using 10% of the sample size (37) respondents from urban and rural communities that were not part of the communities used for the study. The instrument's reliability was confirmed using the Pearson correlation formula to calculate the reliability coefficient, which was established at 0.86.

### Ethical Consideration

Ethical clearance was obtained from the Ministry of Health Research Ethics Committee of Delta State. The reference number is HC218/Vol.11/95. The purpose of the study was explained to the respondents. Written informed consent was obtained from participants before copies of the questionnaire were administered. The choice of respondents to participate and confidentiality were respected.

### Data Collection

Data was collected with the help of eight (8) research assistants. These research assistants were trained on the purpose and objectives of the study and how to administer the questionnaire to the respondents. The questionnaires were administered to the respondents after informed consent was obtained. Three (3) research assistants were assigned to the collection of data from women who were born between April 2015 and December 2015 in Ughelli town, and two (2) research assistants in Ewu, all in Delta Central, while in Delta South, two (2) research assistants were involved in collection of data from women in Ozoro and one (1) research assistant was involved in collection of data from women in Ofagbe. The researcher identified the index house where the first respondent was selected and spun in the estimated centre of the communities. Then, the direction of the bottle after being rotated determined the starting point of sampling of the next household, which respondents will be selected using purposive. Data were collected within 6months period between April 2015 to December 2015

### Data Analysis

All data were entered into Microsoft Excel and then exported to Statistical Package for Social Sciences version 26.0. Frequencies and percentages were the descriptive statistics used for the study. Principal Component Analysis (PCA) in SPSS was also used to compute Socioeconomic Status (SES). The input variables to the PCA were information on key access to water, sanitation, ownership of a radio, bicycle, motorcycle, and refrigerator, model of house and weekly household cost of food, which is strongly related to socioeconomic status. The SES index was used to divide the respondents into five socioeconomic quintiles, from poorest (quintile 1) to wealthiest SES (quintile 5).

The final measure of inequity was the concentration curves.^22^ The concentration curve plots the cumulative proportion of the individuals under consideration ranked by wealth (wealth is the availability of assets and household expenditure) against the cumulative proportion of health/healthcare variables (maternal care utilisation, under–five immunisation) being measured. If there was no wealth-related inequity in the rate of maternal care utilisation, the concentration curve coincided with the diagonal line (line of equity), implying no disparities in FMCHS. However, if the utilisation of the FMCHS is disproportionately higher among those with higher SES, geographical location, and educational status, the concentration curve lies below the equity line.

The concentration index computed from the concentration curve assumed values between −1 and +1. Its value was negative when the concentration curve was above the diagonal and positive when the curve was below the diagonal in the absence of imbalance (the concentration curve coinciding with the diagonal); the value of the concentration index is zero. For grouped data, the concentration index (C) is computed using the following formula: C = (P_1_L_2_ − P_2_L_1_) + (P_2_L_3_ − P_3_L_2_) + … + (P_r_ − L_r_ − P_r_L_r - 1_) 23 where Pr is the cumulative per cent of the sample ranked by economic status, geographical location, and educational status, while Lr is the corresponding cumulative percentage of Health variables; r is the rth quintile. Inferential statistics, including One-way analysis of variance and t-test, were also used to analyse the data so that statistical inference could be made on the population.

## Results

Table 1 shows the socio-demographics of respondents. Most respondents were aged between 21 and 30.

**Table 1:** Socio-demographics of respondents

VARIABLES	f(%), n=368
Age category	
≤ 20years	18(4.9)
21-30Years	217(59)
31-40Years	123(33.4)
>40Years	10(2.7)
**Age. Mean (SD)**	30.167±(5.887)

The respondent went to school	360(97.8)

Highest level of education	
Primary school	63(17.1)
Secondary school	199(54.1)
Tertiary level	106(28.8)

Occupation of respondents	
Civil Servant (Working in Government Establishment)	52(14.1)
Employed private professional	57(15.5)
Self-employed professional	86(23.4)
Farmer	50(13.6)
Petty trading	95(25.8)
Big business owner	10(2.7)
Unemployed	18(4.9)

Whether respondents are married	
Yes	344(93.4)
No	24(6.5)

**Marital status of respondents**	
Single	24(6.5)
Married	327(88.8)
Widowed	7(1.9)
Divorced/separated	10(2.7)

**Average number of years spent schooling–mean(SD)**	12.163±(3.573)

SES Index	
Quintile 1 (Most poor)	76(20.7)
Quintile 2 (Poor)	68(18.5)
Quintile 3 (Average)	74(20.1)
Quintile 4 (Least poor)	76(20.7)
Quintile 5 (rich)	74(20.1)

Most (97.8%) of the respondents had some form of education and were similar across the geographical area. 25.8% of the respondents stated they were petty traders, while 23.3% stated they were self-employed professionals. 15.5%were professionals employed in the private sector, and 14.1% were civil servants working in government establishments, and this was statistically different across the geographical areas. 88.9% of the respondents were married, and this was similar across the geographical areas. About 20% of the respondents belonged to the poorest, poor and least poor SES groups, and about 19.8% belonged to the average poor and rich SES groups.

Stratification of SES was done using Principal Component Analysis (PCA) in SPSS, which was also used to compute Socio-Economic Status (SES). The input variables to the PCA were information on key access to water, sanitation, ownership of a radio, bicycle, motorcycle, refrigerator, model of house and weekly household cost of food, which is strongly related to socioeconomic status. The SES index divided the respondents into five socioeconomic quintiles, from the poorest (quintile 1) to the wealthiest SES (quintile 5).

Table 2 shows that about 21% of the respondents in the second and fourth quintiles have used FMCH, 20% from the fifth quintile have used FMCH, and 18.8% in the first quintile have used FMCH. These are similar across the SES groups at p=0.151. The table also shows statistical similarity across the different educational levels in utilising FMCH at p=0.866 and a statistical difference across the geographic location at p=0.001.

**Table 2:** Level of utilisation of MCH services under the FMHCP among different SES group, educational background and geographical locations

Variables	Yes f(%)	No f(%)	Total f(%)	χ^2^	p-value
**SES**					
**Quintile 1 (Most poor)**	67(88.2)	9(11.8)	76 (100.0)	7.007	0.135
**Quintile 2 (Poor)**	66(97.1)	2(2.9)	68 (100.0)		
**Quintile 3 (Average)**	70(94.6)	4(5.4)	74 (100.0)		
**Quintile 4 (Least poor)**	73(96.1)	3(3.9)	76 (100.0)		
**Quintile 5 (rich)**	69(93.2)	5(6.8)	74 (100.0)		
**Level of Education**					
**Primary school**	60(95.2)	3(4.8)	63 (100.0)	0.288	0.866
**Secondary school**	186(93.5)	13(6.5)	199 (100.0)		
**Tertiary level**	99(93.4)	7(6.6)	106(100.0)		
**Occupation of Respondents**					
**Civil Servant**	51(98.1)	1(1.9)	52(100.0)	4.071	0.667
**Employed private professional**	53(93.0)	4(7.0)	57(100.0)		
**Self-employed professional**	81(94.2)	5(5.8)	86(100.0)		
**Farmer**	45(90.0)	5(10.0)	50(100.0)		
**Petty trading**	90(94.7)	5(5.3)	95(100.0)		
**Big business owner**	9(90.0)	1(10.0)	10(100.0)		
**Unemployed**	16(88.9)	2(11.1)	18(100.0)		
**Geographical Locations**					
**Urban**	260(96.3)	10(3.7)	270(100.0)	11.219	0.001
**Rural**	85(86.7)	13(13.3)	98(100.0)		

Table 2 also shows that the concentration index amongst the educational status is 0.027, implying that there is equity in access amongst the educational group, reflecting the curve below the concentration curve (see Figure 2). Similarly, the F-ratio of 1.010, p > 0.05 indicates that irrespective of the educational status of women, they all have equal access to free maternal and child health services. Contrarily, Table 2 shows that the concentration index between the geographical locations is -0.020, implying disproportionate access between the geographical regions. This means that urban dwellers have more access to free maternal and child health services in Delta State than their counterparts in rural areas. This reflects the concentration curve above the equity line, as shown in Figure 3.

Further, the t-test value of -3.780, p = <0.001, indicates women residents in different locations (urban or rural) do not have equal access to free maternal and child health services. The negative t-value implies that women in urban settings have better/more access to free maternal and child health services than women in rural settings.

Table 3 shows that the concentration index between the socioeconomic groups is 0.014, reflecting a positive concentration index and indicating equity of access to free maternal and child health services amongst the socioeconomic groups. However, the fourth SES group has a negative concentration, indicating disproportionate access.

**Table 3:** Inequity in access using concentration index to FMCH between SES status, geographical location and educational level

SES Index	N (FMCH	Rel %	C1	C2	C.Index	F-ratio	P-level
Quintile 1 (Most poor)	65	18.8	0.079	0.076	0.0030	1.170	0.120
Quintile 2 (Poor)	71	20.6	0.242	0.237	0.0050		
Quintile 3 (Average)	68	20.7	0.486	0.482	0.004		
Quintile 4 (Least poor)	72	20.8	0.802	0.8	-0.002		
Quintile 5 (rich)	69	20.0			0.000		
					0.014		

Level of education						1.010	0.140
Primary	60	0.215	0.110	0.153	-0.04		
Secondary	120	0.430	0.712	0.645	0.069		
Tertiary	99	0.355			0.0000		
					0.027		

Geographical area						t-value = -	0.000*
Urban	260	260	0.734	0.754	0.0200	3.780	
Rural	86	24.6			0.0000		
					-0.0200		

* significant @ 0.05 level

**Figure 1:**
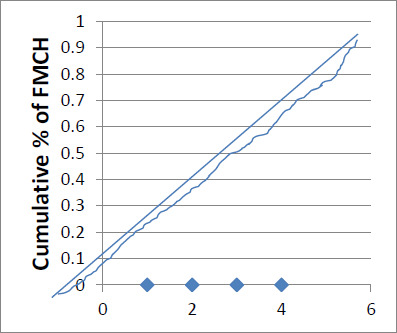
Concentration curve for SES and FMCH

This implies that by plotting this in a concentration curve, the curve will lie below a 45-degree diagonal line, as in Figure 1. Similarly, the F-ratio of 1.170, p > 0.05 indicates that irrespective of the socioeconomic status of women, they all have equal access to free maternal and child health services; therefore, the null hypothesis which states that there is no significant difference between socioeconomic status of women and access to free maternal and child health services was retained while the alternate hypothesis was rejected.

**Figure 2:**
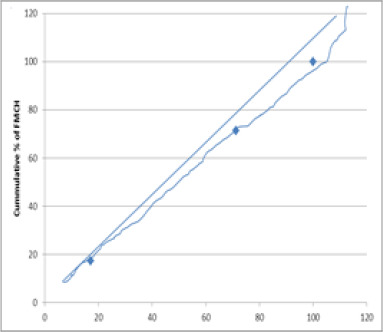
Concentration curve for educational level and FMCH

**Figure 3:**
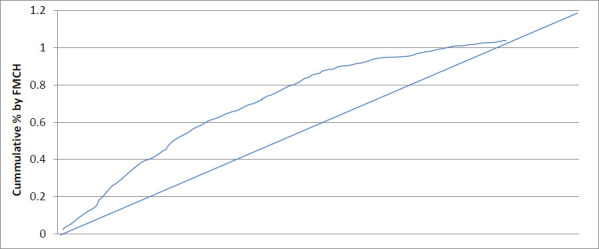
Showing concentration curve for geographical location and FMCH

Figure 1 indicates equity among the SES and FMCH because the calculated concentration index was positive, and the curve lies below the equity line. Figure 2 indicates equity among different educational statuses because the calculated concentration index was positive, and the curve lies below the equity line. Figure 3 indicates that there is an inequity between the geographical location and FMCH because the calculated concentration index was negative, and the concentration curve lies above the equity line.

## Discussion

Findings from the study showed that most respondents were aged between 21 years and 30 years. The majority (97.8%) had some form of education, and this was similar across the geographical areas. This was due to primary and secondary schools in most villages following the introduction of free education in 1979 during the Unity Party free education policy and the attendant expansion of schools in most communities in Bendel State, from which Delta State was created. Overall, this signifies that the level of literacy is high in Delta State. 23.3% stated that they were self-employed professionals, and only 14.1% were civil servants working in government establishments, and this is statistically different across the geographical areas. Notably, most women were married and living together with their spouses, which was similar across the geographical areas. Only a few (19.8%) belong to the average poor and rich SES groups. Findings from the utilisation of free maternal and child health services revealed that the second (poor) and fourth (least poor) quantiles utilised the services more. This is at variance with the studies conducted in India and Namibia^10^,^12^, which revealed that the urban and rich quantiles utilised health services more.

This could be because there is more awareness in urban than rural areas. Interestingly, the findings showed equity between different SES groups and educational backgrounds. The equity found in this study may be due to the free nature of the intervention, whereby all SES groups could afford the services because of the free nature of services. These findings are at variance with a study conducted in Niger state, Nigeria, which revealed that health status and education are major drivers of inequity^13^.

Also, other studies showed that there is persistent pro-rich inequities in access to four maternal health interventions in both Brazil and Columbia.^9^, ^12^.

However, there was geographic inequity because the urban dwellers used the services much more than the rural dwellers. This could be due to distance (physical access) between respondents' homes and the hospital in the rural area. This aligns with the study in Ghana, where geographical location, income status and formal education are key drivers of maternal and child health inequities^11^. A study conducted in India supported these findings, revealing a significant disparity between urban residents and the richest quintile and rural residents and the poorest quintile.^10^

The government should find ways of improving access by making services geographically accessible to all groups. This can be done by making transportation affordable to reproductive-aged women who are willing to access free maternal and child health services. This study is relevant to all healthcare professionals because it will encourage them, especially nurses, to exercise their energy towards home care to reduce maternal and child mortality. This is done by encouraging community midwifery where these women would be cared for at home, especially for those who cannot access the hospital since distance is a strong determinant of access in rural areas. This would go a long way to reduce maternal and child mortality, which is the primary aim of free maternal and child services.

The study's limitations stem from data collected from household surveys based on self-reporting, which needed to be independently verified.

There may be a possibility of recall bias or wilful statements, which may invalidate the findings.

## Conclusion

Equity was found in using the FMHCS between different SES groups and those with different educational levels. However, there was inequity between urban and rural areas in favour of urban dwellers, possibly due to the distance between rural dwellers and the hospitals. Since free maternal and child health services were introduced, no study has been done to establish equitable access to the services. The gap in the study revealed that distance was a strong determinant of access to free maternal and child health services.
